# Supplementary material for: Inferring tumor progression in large datasets
Source: PLoS Comput Biol. 2020 Oct 9;16(10):e1008183. doi: 10.1371/journal.pcbi.1008183 (PMC7577444; doi:10.1371/journal.pcbi.1008183)
Supplement: S1 Text — (PDF) [file pcbi.1008183.s001.pdf]

Inferring Tumor Progression in Large Datasets: Supporting Information 1

Mohammadreza Mohaghegh Neyshabouri, Seong-Hwan Jun, Jens Lagergren

Likelihood calculation details

We want to calculate $p(Y|\mathcal{P}, \sigma_{1:M}, \epsilon, \delta)$, where $Y = \{Y_m : m \in \{1, \ldots, M\}\}$ is our observation matrix, $\sigma_{1:M} = (\sigma_1, \ldots, \sigma_M)$ is the given vector of progression stages of the tumors, and $\mathcal{P} = (D_1, D_2, \ldots, D_L, P)$ is our pathway progression model. Since the tumors are independent given $\mathcal{P}$ and $\sigma_{1:M}$,

$$p(Y|\mathcal{P}, \sigma_{1:M}, \epsilon, \delta) = \prod_{m=1}^{M} p(Y_m|\mathcal{P}, \sigma_m, \epsilon, \delta).$$

We separate the bits corresponding to different pathways as:

$$p(Y_m|\mathcal{P}, \sigma_m, \epsilon, \delta) = p(Y_{m,P}|\mathcal{P}, \sigma_m, \epsilon, \delta) \prod_{l=1}^{L} p(Y_{m,D_l}|\mathcal{P}, \sigma_m, \epsilon, \delta) \quad (1)$$

In order to calculate each term of form $p(Y_{m,S}|\mathcal{P}, \sigma_m, \epsilon, \delta)$ in (1), we marginalize over all possible noise-free vectors $Y^*_{m,S}$:

$$p(Y_{m,S}|\mathcal{P}, \sigma_m, \epsilon, \delta) = \sum_{Y^*_{m,S}} p(Y^*_{m,S}|\mathcal{P}, \sigma_m) p(Y_{m,S}|Y^*_{m,S}, \epsilon, \delta) \quad (2)$$

If $S \in \{D_1, \ldots, D_{\sigma_m}\}$, $Y^*_{m,S}$ has to be a one-hot binary vector of length $|S|$. Denoting the number of ones in the observed $Y_{m,S}$ by r, we have:

$$p(Y_{m,S}|\mathcal{P}, \sigma_m, \epsilon, \delta) = \frac{r}{|S|}(1-\delta)\epsilon^{r-1}(1-\epsilon)^{|S|-r} + \frac{|S|-r}{|S|}\delta\epsilon^{r}(1-\epsilon)^{|S|-r-1}. \quad (3)$$

The first summand in this expression corresponds to the probability of the 1 in the latent $Y^*_{m,S}$ being among our r observed 1's in $Y_{m,S}$ (which is the case with probability of $r/|S|$), times the probability of getting to $Y_{m,S}$ from such a $Y^*_{m,S}$. In this case, $Y_{m,S}$ is obtained by the 1 in $Y^*_{m,S}$ being kept from flip-back, followed by passenger mutations in $r-1$ genes (leading to the total of r observed mutations) and no false positives in the remaining $|S|-r$ genes. Similarly, the second summand in (3) corresponds to the probability of the 1 in the latent $Y^*_{m,S}$ being among our $|S|-r$ observed 0's in $Y_{m,S}$ (due to a flip-back). This is the case with probability of $(|S|-r)/|S|$, and if it is, then $Y_{m,S}$ is obtained by a flip-back, followed by passenger mutations in r genes and no false positives in the remaining $|S|-r-1$ genes.

If $S \in \{D_{\sigma_m+1}, \ldots, D_L\}, P$, $Y^*_{m,S}$ has to be a vector of $|S|$ zeros. Hence, observing r ones in $Y_{m,S}$, we have exactly r false positives, leading to:

$$p(Y_{m,S}|\mathcal{P}, \sigma_m, \epsilon, \delta) = \epsilon^{r}(1-\epsilon)^{|S|-r}. \quad (4)$$

Algorithm S1. Fast calculation of the likelihood $p(Y|\mathcal{P}, \alpha, \epsilon, \delta)$

Initialize $\mathcal{A}$ and $\mathcal{B}$ to be zero matrices of shape (z, z), where $z = \max_{l\in[L]} |D_l|$
for all $m \in \{1, \dots, M\}$ **do** ▷ Calculate $p(Y_m|\mathcal{P}, \alpha, \epsilon, \delta)$
  for $\sigma_m \in \{1, \dots, L\}$ **do** ▷ Calculate $p(Y_m, \sigma_m|\mathcal{P}, \alpha, \epsilon, \delta)$
    $R \leftarrow 1$
    for $S \in \{D_1, ..., D_L, P\}$ **do**
      $r = \|Y_{m,S}\|_1$
      if $S \in \{D_1, ..., D_{\sigma_m}\}$ **then**
        if $\mathcal{A}\big[|S|, r\big] == 0$ **then**
          $A = \frac{r}{|S|}(1-\delta)\epsilon^{r-1}(1-\epsilon)^{|S|-r} + \frac{|S|-r}{|S|}\delta\epsilon^{r}(1-\epsilon)^{|S|-r-1}$
          $\mathcal{A}\big[|S|, r\big] \leftarrow A$
        else
          $A = \mathcal{A}\big[|S|, r\big]$
      else
        if $\mathcal{B}\big[|S|, r\big] == 0$ **then**
          $A = \epsilon^{r}(1-\epsilon)^{|S|-r}$
          $\mathcal{B}\big[|S|, r\big] \leftarrow A$
        else
          $A = \mathcal{B}\big[|S|, r\big]$
      $R \leftarrow R * A$
    $p(Y_m|\mathcal{P}, \sigma_m, \epsilon, \delta) = R$
    $p(Y_m, \sigma_m|\mathcal{P}, \alpha, \epsilon, \delta) = p(\sigma_m|\alpha)p(Y_m|\mathcal{P}, \sigma_m, \epsilon, \delta)$
  $p(Y_m|\mathcal{P}, \alpha, \epsilon, \delta) = \sum_{\sigma_m=1}^{L} p(Y_m, \sigma_m|\mathcal{P}, \alpha, \epsilon, \delta)$
$p(Y|\mathcal{P}, \alpha, \epsilon, \delta) = \prod_{m=1}^{M} p(Y_m|\mathcal{P}, \alpha, \epsilon, \delta)$

Unknown progression stages

In practice, we do not have the progression stages of individual tumors. Fortunately, we can marginalize out the progression stages vector $\sigma_{1:M}$ using the independence assumption over the samples given the pathways:

$$p(Y|\mathcal{P}, \alpha, \epsilon, \delta) = \sum_{\sigma_{1:M}} p(Y, \sigma_{1:M}|\alpha, \mathcal{P}, \epsilon, \delta) = \prod_{m=1}^{M} \left(\sum_{\sigma_m=1}^{L} p(Y_m, \sigma_m|\mathcal{P}, \alpha, \epsilon, \delta) \right) \tag{5}$$

In order to calculate $p(Y_m, \sigma_m|\mathcal{P}, \alpha, \epsilon, \delta)$, we can write it as

$$p(Y_m, \sigma_m|\mathcal{P}, \alpha, \epsilon, \delta) = p(\sigma_m|\alpha)p(Y_m|\mathcal{P}, \sigma_m, \epsilon, \delta), \tag{6}$$

where the first term is the prior belief on the stages being σ_m and the second term is given by (1). We consider a uniform prior on the progression stages $\sigma_{1:M}$, i.e., $\alpha = (1/L, \dots, 1/L)$. However, an alternative prior can be chosen.

In the following subsection, we describe a systematic likelihood calculation scheme, which can prevent us from repetitive calculations while going over our M tumors in the data.

Fast Likelihood Calculation

Given a progression model $\mathcal{P} = (D_1, \ldots, D_L)$, and the data matrix Y, we form a matrix C of shape (M, L), where $C_{i,j}$ is the number of mutations of tumor i in driver pathway j. Denoting the size of our largest pathway by $z = \max_{l \in [L]} |D_l|$, we form two look-up tables in form of zero matrices $\mathcal{A}$ and $\mathcal{B}$ of shape (z, z). We modify our likelihood calculation algorithm to check the lookup tables before any repetitive calculations. The modified procedure is provided in Algorithm S1.
