# Supplementary material for: Inferring tumor progression in large datasets
Source: PLoS Comput Biol. 2020 Oct 9;16(10):e1008183. doi: 10.1371/journal.pcbi.1008183 (PMC7577444; doi:10.1371/journal.pcbi.1008183)
Supplement: S2 Text — (PDF) [file pcbi.1008183.s002.pdf]

Inferring Tumor Progression in Large Datasets: Supporting Information 2

Mohammadreza Mohaghegh Neyshabouri, Seong-Hwan Jun, Jens Lagergren

Model selection details

The posterior probability of a model length $L \in \mathcal{L}$ given the observation can be written as

$$p(L|Y) = \frac{p(Y|L)p(L)}{\sum_{L \in \mathcal{L}} p(Y|L)p(L)}.$$

Our uniform prior on the model length corresponds to setting $p(L) = 1/|\mathcal{L}|$, hence, $p(L|Y) \propto p(Y|L)$. Equation (3) of the paper can be simply derived as follows,

$$\mathbb{E}_{p(\mathcal{P},\epsilon,\delta|Y,L)}\left[\frac{1}{p(Y|\mathcal{P},\epsilon,\delta,L)p(\epsilon,\delta)p(\mathcal{P}|L)}\right] = \int \frac{p(\mathcal{P},\epsilon,\delta|Y,L)}{p(Y|\mathcal{P},\epsilon,\delta,L)p(\epsilon,\delta)p(\mathcal{P}|L)} d\mathcal{P}d\epsilon d\delta = \frac{1}{p(Y|L)}$$

The MCMC estimate of the LHS in this equation will be

$$\mathbb{E}_{p(\mathcal{P},\epsilon,\delta|Y,L)}\left[\frac{1}{p(Y|\mathcal{P},\epsilon,\delta,L)p(\epsilon,\delta)p(\mathcal{P}|L)}\right] \approx \frac{1}{I}\sum_{i=1}^{I} \frac{1}{p(Y|\mathcal{P}_i,\epsilon_i,\delta_i,L)p(\epsilon_i,\delta_i)p(\mathcal{P}_i|L)}.$$

One potential problem is in the computation of $p(\mathcal{P}_i|L)$, where we need to calculate the cardinality of the space of valid pathway progression models of length L: $|\mathcal{X}(L)|$. Although enumeration over $\mathcal{X}(L)$ appears intractable, there is a closed-form formula for computing its cardinality, $|\mathcal{X}(L)|$. Given a set of N genes, a valid progression of length L consists of L non-empty driver pathways and a set of passenger genes. Let $f_N(L)$ denote the number of valid ways to allocate N genes to L non-empty driver pathways and the set of passengers (which can remain empty). We can calculate $f_N(L)$ using the recursive formula

$$f_N(L) = (L+1)^N - \sum_{i=1}^{L} \binom{L}{i} f_N(L-i).$$

Note that $f_N(L)$ is the total number of possible assignments, $(L+1)^N$, minus the number of invalid assignments. We count the number of invalid assignments with i empty driver pathways separately. There exist $\binom{L}{i}$ different choices for a set of i driver pathways to keep empty. For each case, we can have $f_N(L-i)$ different valid assignment of genes to the remaining pathways, ensuring that none of the $L-i$ remaining driver pathways are empty.
