# Supplementary material for: Inferring tumor progression in large datasets
Source: PLoS Comput Biol. 2020 Oct 9;16(10):e1008183. doi: 10.1371/journal.pcbi.1008183 (PMC7577444; doi:10.1371/journal.pcbi.1008183)
Supplement: S3 Text — (PDF) [file pcbi.1008183.s003.pdf]

Inferring Tumor Progression in Large Datasets: Supporting Information 3

Mohammadreza Mohaghegh Neyshabouri, Seong-Hwan Jun, Jens Lagergren

Details of synthetic data simulations

POCO: a performance metric for synthetic data experiments

We introduce a performance metric named *POCO* (standing for the percentage of correct ordering of genes) for our synthetic data simulations. We use this metric to assess the similarity of our inferred progression model $\hat{\mathcal{P}}$ to the known generative model $\mathcal{P}$. We calculate the *POCO* score of $\hat{\mathcal{P}}$ with respect to $\mathcal{P}$ as follows. Let N be the number of genes in $\mathcal{P}$ and $\hat{\mathcal{P}}$. We go over all gene pairs (g_1, g_2) and check if their respective order in $\mathcal{P}$ (g_1 before g_2, g_1 in the same set as g_2, or g_1 after g_2) is preserved in $\hat{\mathcal{P}}$. The POCO score will be the fraction of these gene pairs with correct respective order in $\hat{\mathcal{P}}$. Note that we stick the set of passengers to the end of the progression model such that all genes in the driver pathways are considered as placed before the genes in the set of passengers.

As various methods for progression model inference might use different optimality criteria, POCO enables us to compare the performances. The main limitations of the metric can be summarized as follows. Firstly, while errors in the first driver pathways are considered more significant from a biological point of view, POCO implicitly considers the same weights for all pairs of genes. Moreover, considering only the respective positions of the pairs of genes may become problematic. A worst-case example would be to have an inferred model where a driver pathway including a single passenger gene is added to the beginning of the progression model. While such an inferred model has a critical error, its POCO score will be relatively high as most of the respective orders are preserved. However, despite all such drawbacks, considering the respective order of genes seems to make more biological sense compared to the alternatives such as considering the correct clustering rate of single genes. Fig S1 shows several illustrative examples of common inference errors and their effects on the POCO score. As shown in these examples, POCO is severely affected by misplacing the driver pathways, while dividing a driver pathway into two consecutive driver pathways or merging two adjacent driver sets do not lead to significant drops in the POCO score.

Experiment 1 details

In all the experiments in this section, we have given a 60 seconds time limit to ILP. We have used MCMC with 2500 iterations (first 500 discarded as burn-in phase) with 0.9 probability of *gene-move* and 0.1 probability of *pathway-swap*, 10 Metropolis-Hasting iterations within each for error rate (ϵ or δ) update, and Gaussian random walk proposal for ϵ and δ with standard deviation of 0.05. All MCMC run times were less than 30 seconds.

One of the main advantages of a Bayesian approach such as ours, compared to the ILP algorithm, is the posterior distribution we can generate for the error parameters. Fig S2 shows our performance on estimations of the error parameters. The true (generative) values of the error parameters are shown using the red dotted lines. As shown in this figure, the estimates get more and more precise as the number of patients increases.

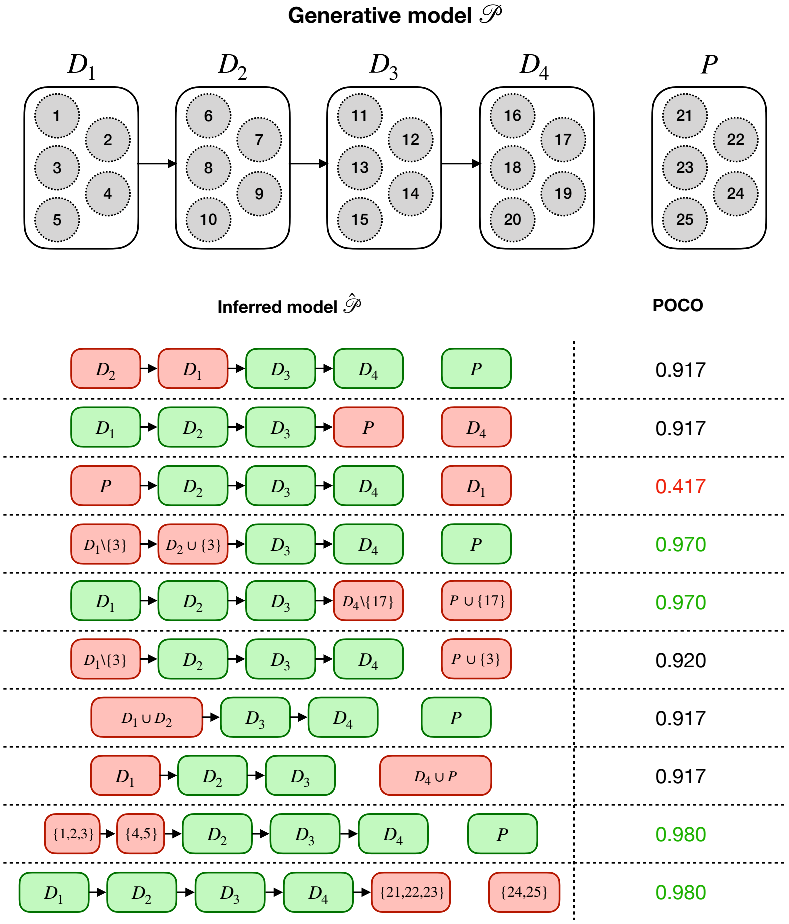

Fig S1. An example generative model and the POCO score of several erroneous inferred models. The pathways shown in green include the correct sets of genes and are placed in the correct position in the progression model, while the ones shown in red are erroneous.

Experiment 2 details

In this experiment, for the MCMC algorithm, we have used the same setting as the previous experiment except for a prior passenger probability of 0.99 (for all genes) and an increment in the number of iterations to 10000 (first 2000 are discarded as burn-in phase). Each ILP run had a time limit of 600 seconds, while the MCMC run times in this setting take less than 60 seconds typically (one-tenth of our ILP time limit).

In Fig S3, we show the precision, recall and F1 score of the algorithms for the task of specific pathway identification. It can be seen that the detection of the genes in the last pathways is a harder task for all the methods, as expected. Our MCMC method outperforms all the ILP algorithms, even the one with the extra knowledge on the generative error parameter (ILP with $\epsilon = 0.05$).

Experiment 3 details

In this experiment, the MCMC parameters are exactly similar to the previous experiment, except for the varying model length. After running MCMC with various model length, we have chosen the inferred model length based on the dataset evidence provided by the MCMC algorithm.

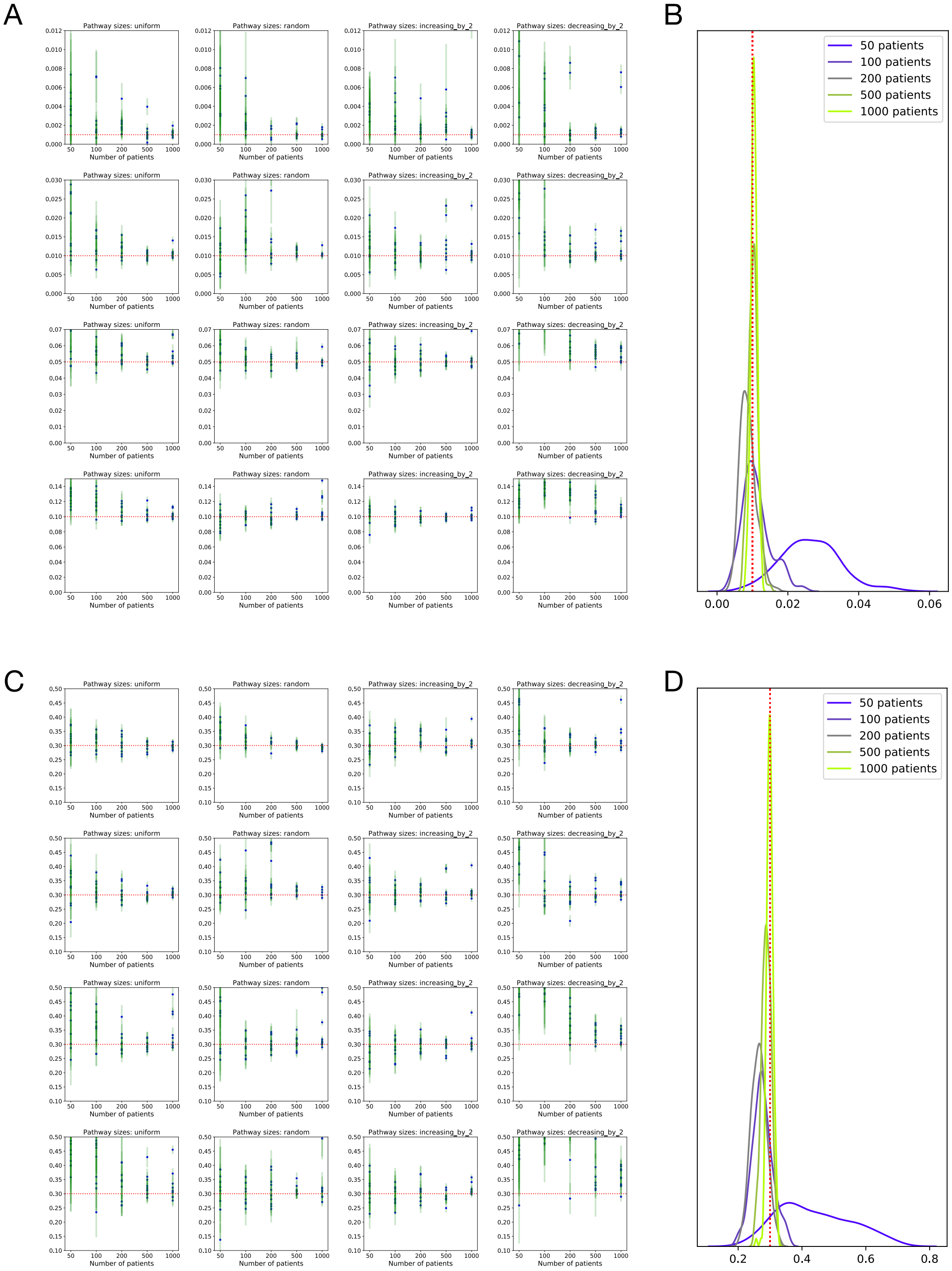

Fig S2. The estimated error parameters for Experiment 1. The red dotted lines show the true generative value for the error parameters. A: The posterior means (blue dots) and standard deviations (green lines) of the background mutation rates for all MCMC chains. B: Example KDEs for the background mutation rates. C: The posterior means (blue dots) and standard deviations (green lines) of the flip-back probabilities for all MCMC chains. D: Example KDEs for the flip-back probabilities.

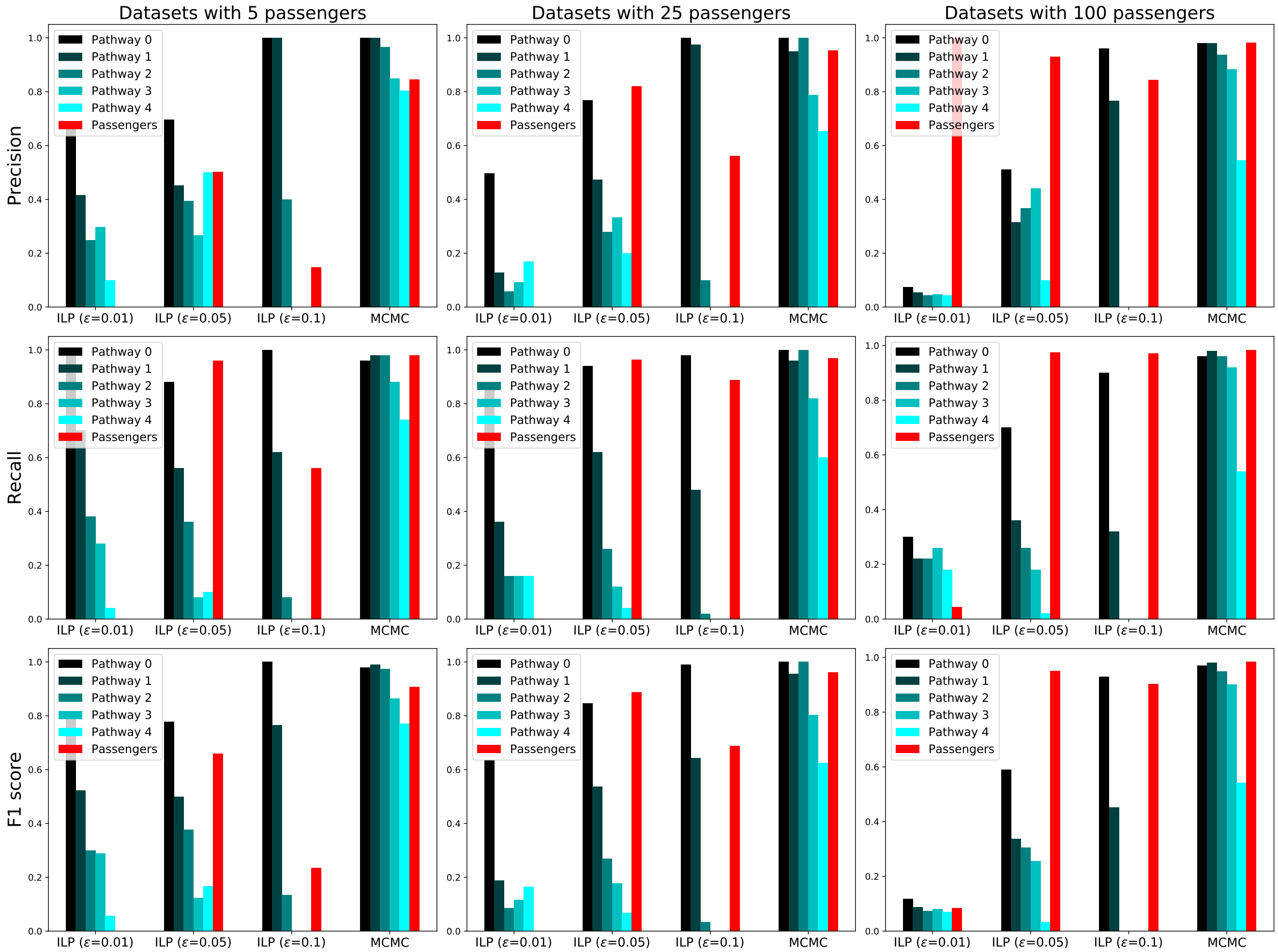

Fig S3. Precision, recall and F1 scores in detection of the genes in pathways 1 to 5 and the set of passengers in Experiment 2.
