# Supplementary material for: Inferring tumor progression in large datasets
Source: PLoS Comput Biol. 2020 Oct 9;16(10):e1008183. doi: 10.1371/journal.pcbi.1008183 (PMC7577444; doi:10.1371/journal.pcbi.1008183)
Supplement: S4 Text — (PDF) [file pcbi.1008183.s004.pdf]

Inferring Tumor Progression in Large Datasets: Supporting Information 4

Mohammadreza Mohaghegh Neyshabouri, Seong-Hwan Jun, Jens Lagergren

Details of biological data analysis

Averaging the MCMC model samples

We use Algorithm S1 to produce a consensus-like model from a set of models collected from the MCMC run. The goal of this algorithm is to output a progression model (of the same length as the input models) that preserves the genes respective orders as much as possible.

More details on our biological data analysis

As the set of potential drivers typically includes less than one percent of the genes, the background mutation rate has to be roughly equal to the mutation rate of the dataset. We used this information to set the cost coefficient of the passenger genes in the ILP-based method and set our algorithm's background mutation rate to be equal to the mutation rate of the dataset.

For our algorithm, we had 10000 MCMC iterations for each model length. We had 10 MH iterations for δ parameter within each Gibbs iteration. After running MCMC with various model lengths, we chose the inferred model length based on the dataset evidence provided by the MCMC algorithm. We used a thinning interval of 10 samples to collect less correlated samples and considered the last 500 samples to compute the consensus-like model as our output.

For the ILP-based method, we considered a time limit of 600 seconds for each ILP run. The cost coefficient of the passenger genes was set using the information on the background mutation rate. The model selection was performed using the procedure suggested by the corresponding publication on the same set of model length candidates as the one used for the MCMC (from length 2 to 30).

Algorithm S1. Mixing MCMC samples from the posterior distribution of the progression models.

Require: matrix of MCMC samples M of shape (k, n), where $M[i, j]$ is the index of pathway including gene j in sample i. We denote the models length by L.

Initialize A, B, C and D to be zero matrices of shape (n, n)
Initialize G to be an empty vector ▷ will includes set of genes in pathways
for all $i \in \{1, \ldots, k\}$ **do**
    for all $j_1 \in \{1, \ldots, n\}$ **do**
        for all $j_2 \in \{j_1 + 1, \ldots, n\}$ **do**
            if $M[i, j_1] > M[i, j_2]$ **then** ▷ gene j_1 is put after gene j_2
                $A[j_1, j_2] \leftarrow A[j_1, j_2] + 1$
                $B[j_2, j_1] \leftarrow B[j_2, j_1] + 1$
            else if $M[i, j_1] < M[i, j_2]$ **then** ▷ gene j_1 is put before gene j_2
                $B[j_1, j_2] \leftarrow B[j_1, j_2] + 1$
                $A[j_2, j_1] \leftarrow A[j_2, j_1] + 1$
            else ▷ the genes are put together
                $C[j_1, j_2] \leftarrow C[j_1, j_2] + 1$
                $C[j_2, j_1] \leftarrow C[j_2, j_1] + 1$
for all $j_1 \in \{1, \ldots, n\}$ **do**
    for all $j_2 \in \{1, \ldots, n\}$ **do**
        if $A[j_1, j_2] > B[j_1, j_2]$ **then**
            $D[j_1, j_2] \leftarrow D[j_1, j_2] + 1$ ▷ gene j_1 should be an ancestor of gene j_2
Let x be the row sum of D ▷ $x[j]$ is the number of descendants of gene j
$u = \text{Sort}(x)$
for all $v \in u$ **do**
    Append the set of gene j's with $x[j] = v$ to G
while $|G| > L$ **do** ▷ the output model length should be reduced
    Let R be a matrix of shape $(|G|, |G|)$
    for each pair of groups in G denoted by g_1 and g_2 **do**
        Using C, calculate the rate of genes in g_1 and g_2 being in the same pathway in the MCMC samples and put the resulting rate in $R[g_1, g_2]$
    Merge the two groups with the maximum $R[g_1, g_2]$ value and put the resulting group in place of the latter one among g_1 and g_2.
Return G
